# Evoked Alpha Power is Reduced in Disconnected Consciousness During Sleep and Anesthesia

**DOI:** 10.1038/s41598-018-34957-9

**Published:** 2018-11-09

**Authors:** Matthieu Darracq, Chadd M. Funk, Daniel Polyakov, Brady Riedner, Olivia Gosseries, Jaakko O. Nieminen, Vincent Bonhomme, Jean-Francois Brichant, Melanie Boly, Steven Laureys, Giulio Tononi, Robert D. Sanders

**Affiliations:** 1 Department of Anesthesiology, University of Wisconsin, Madison, 53792 USA; 2 Department of Psychiatry, University of Wisconsin, Madison, 53719 USA; 3 Coma Science Group, GIGA-consciousness, University of Liège, Liège, 4000 Belgium; 4 Department of Neuroscience and Biomedical Engineering, Aalto University School of Science, Espoo, Finland; 5 Anesthesia and Intensive Care Laboratory, GIGA-Consciousness, Liège University, Liège, 4000 Belgium; 6 Department of Anestheisa and ICM, CHU Liège, Liège, 4000 Belgium; 7 University Department of Anesthesia and ICM, CHR Citadelle, Liège, 4000 Belgium; 8 Department of Neurology, University of Wisconsin, Madison, 53792 USA; 9 Department of Neurology, CHU Liège, Liège, 4000 Belgium

## Abstract

Sleep and anesthesia entail alterations in conscious experience. Conscious experience may be absent (unconsciousness) or take the form of dreaming, a state in which sensory stimuli are not incorporated into conscious experience (disconnected consciousness). Recent work has identified features of cortical activity that distinguish conscious from unconscious states; however, less is known about how cortical activity differs between disconnected states and normal wakefulness. We employed transcranial magnetic stimulation–electroencephalography (TMS–EEG) over parietal regions across states of anesthesia and sleep to assess whether evoked oscillatory activity differed in disconnected states. We hypothesized that alpha activity, which may regulate perception of sensory stimuli, is altered in the disconnected states of rapid eye movement (REM) sleep and ketamine anesthesia. Compared to wakefulness, evoked alpha power (8–12 Hz) was decreased during disconnected consciousness. In contrast, in unconscious states of propofol anesthesia and non-REM (NREM) sleep, evoked low-gamma power (30–40 Hz) was decreased compared to wakefulness or states of disconnected consciousness. These findings were confirmed in subjects in which dream reports were obtained following serial awakenings from NREM sleep. By examining signatures of evoked cortical activity across conscious states, we identified novel evidence that suppression of evoked alpha activity may represent a promising marker of sensory disconnection.

## Introduction

Conscious experience is reversibly altered by sleep and anesthesia. During deep non-rapid eye movement (NREM) sleep or following administration of anesthetics such as propofol, consciousness may be transiently abolished. In other states, such as rapid eye movement (REM) sleep and ketamine anesthesia, conscious experience occurs but most external stimuli are not incorporated into what the subject is experiencing. The existence of such states of “disconnected consciousness”^1^ indicates that the neural mechanisms that construct conscious experience are distinct from the mechanisms that enable physical stimuli to drive conscious content linked to the environment.

Alterations in conscious state reflects underlying changes in cortical activity modes. In wake, the presence of neuromodulators such as noradrenaline and acetylcholine leads to tonic depolarization and irregular neuronal firing, and the electroencephalography (EEG) is said to be “activated”, with high-frequency, low-amplitude activity. In contrast, in deep NREM sleep, neurons oscillate between periods of depolarization with strong firing and periods of hyperpolarization with neuronal silence. The EEG is dominated by high-amplitude, low-frequency “slow waves”. Substantial evidence indicates that states in which the cortex is mostly “activated” are associated with conscious experience, while those in which slow-wave activity is the dominant mode are associated with unconsciousness^2^. Several recent studies using transcranial magnetic stimulation (TMS) combined with EEG have elucidated the consequences of these activity modes on cortical interactions. When the cortex is stimulated with TMS pulses during states in which conscious experience is diminished, including deep NREM sleep, propofol anesthesia and in patients with an unresponsive wakefulness syndrome/vegetative state, EEG responses are stereotyped and lack complexity^3,4^. Moreover, cortical effective connectivity is reduced^5^. This is thought to be due to the occurrence of OFF periods, presumably because the abrupt neuronal silence disrupts cortical communication^6^. Instead, in conscious states including wake, REM sleep, and ketamine anesthesia, cortical responses exhibit rich spatiotemporal responses that persist in time and space, reflecting a capacity for entering a large repertoire of cortical states that is likely a prerequisite for consciousness^4,5^.

These and other studies have established common features of conscious states, but the question of how cortical function differs between disconnected and connected states remains. Cortical activity during REM sleep is classically thought to be wake-like, although recent evidence demonstrates that local cortical^7^ and thalamic^8^ regions unexpectedly exhibit slow-wave activity. Ketamine anesthesia also shows some increased slow-wave activity compared to wake^4^, although the levels are modest relative to anesthetics that lead to unconsciousness. In order to understand how sensory input does not trigger conscious experiences in disconnected states, it is necessary to identify the fundamental ways in which cortical activity differs from awake consciousness in disconnected states.

The fate of incoming stimuli – whether or not they trigger a conscious percept – is partially determined by ongoing cortical activity at the time that stimulus-evoked feedforward activity reaches a given cortical area. Studies of electrophysiological activity at the time of stimulus presentation have shown that activity in the alpha band in awake subjects regulates whether liminal stimuli are perceived^9–12^, suggesting that alpha activity may be particularly important in awake consciousness. Furthermore, evoked widespread alpha-band synchronization accompanies the perception of sensory stimuli during wakefulness^13^ and facilitates connectivity of sensory networks^14^. A natural question, then, is whether changes in alpha activity may also be involved in the characteristic sensory disconnection of states such as sleep and anesthesia, especially when consciousness is preserved in the form of dreaming^1,15^. In this context, it is intriguing that a reduction of spontaneous alpha activity has long been considered a signature of falling asleep^16^, and more recently, anesthesia^17^. During the transition to sleep (N1 in the current nomenclature) subjects become disconnected from the environment, yet if awakened they typically report hallucinatory experiences, known as hypnagogic hallucinations. Thus, the reduction of spontaneous alpha activity upon falling asleep may reflect changes in the excitability of corticothalamic network that are essential for environmental stimuli to trigger conscious perception. In addition, a recent study of steady-state visual evoked potentials identified a decrease in evoked responses in the alpha range during the disconnected state of REM sleep^18^, raising the possibility that the mechanisms that generate alpha oscillations may be dampened in all disconnected states. Hence we hypothesized that evoked alpha power could be a marker of sensory disconnection.

In this study, we analyzed data from previous research studies where TMS–EEG was used to study the reactivity of posterior cortex across a range of behavioral states, including wake, NREM and REM sleep, and propofol and ketamine anesthesia. TMS–EEG provides a unique insight into cortical function, as it may reveal the underlying cortical responsiveness to perturbation that may not be apparent in resting-state EEG responses^19^. Importantly, TMS may reveal the underlying tendency of networks to oscillate in specific frequencies^20^ providing critical information about the network changes involved in transitions across states. Herein we identify an evoked spectral signature of disconnected states, namely, reduced evoked alpha activity.

## Methods

The protocol was approved by the local ethics committee of the University of Wisconsin–Madison and the Medicine Faculty of the University of Liege. The research was performed within the relevant guidelines/regulations and informed consent was obtained from all subjects involved. Much of the data analyzed have been previously reported^3,4,21^. Here, we report hypothesis driven secondary analyses about the signatures of unconsciousness, defined as states associated with reports of “no experience”, and disconnected consciousness, defined as states with dream or dream-like reports with conscious experience not driven by the external world (“sensory disconnection”).

### Anesthesia Studies

Data are reported from the propofol and ketamine datasets published previously^3,4^. Data from an additional anesthetic– xenon^3^ – were not analyzed for the present report because there was an insufficient number of subjects with parietal-targeted TMS. Following written informed consent, subjects underwent parietal TMS–EEG (~110 V/m) during wakefulness at the University of Liege. In eight subjects, an intravenous catheter was placed for target-controlled infusion (TCI Marsh model, Alaris TIVA, CareFusion) delivery of propofol supervised by a certified anesthesiologist. The initial plasma concentration target for propofol was set at 3 μg/ml. This was then adjusted to achieve a state of unresponsiveness to verbal command or mild shaking (Ramsay sedation score of 5–6). The Ramsay sedation scale was assessed every five minutes. Once the desired level of sedation was achieved, TMS–EEG recordings were begun after a five-minute equilibration period (allowing propofol concentration to equilibrate between body compartments). Throughout the experiment, oxygen was administered through a loosely fitted facemask. None of the subjects recalled events after recovery from propofol-induced unresponsiveness, suggesting that a state of unconsciousness was achieved. In separate experiments, six subjects received ketamine. Ketamine was also titrated by TCI (Domino model) supervised by a certified anesthesiologist and titrated to a Ramsay sedation score of 5–6. Following another five-minute equilibration period, TMS–EEG was conducted. In contrast to the unconscious state induced by propofol, retrospective report from subjects under ketamine suggested vivid dream-like experiences consistent with disconnected consciousness. The reported experiences were not driven by the physical environment of the participant.

### Sleep studies

NREM- and REM-sleep data were collected during a previous study^21^. In brief, seven healthy subjects underwent TMS during wake and sleep. All participants gave written informed consent and the experiment was approved by the University of Wisconsin Human Subjects Committee. They underwent five nights of laboratory sleep (divided into 3 and 2 night blocks) with TMS–EEG experiments collected over five nights. Sleep stage was scored manually in 30-second epochs by polysomnography-trained sleep scorers according to the American Academy of Sleep Medicine Scoring Manual. Data were analyzed from epochs identified as REM and N2/N3 NREM sleep. The prevalence of reported conscious experience varies with state. On average, unconsciousness is reported in the majority of arousals from NREM sleep (58% from N2 and 77% from N3). In contrast, 82% of arousals from REM sleep are associated with dreams which is disconnected consciousness^22^. Because unconsciousness and disconnection are the predominant states in NREM and REM sleep, respectively, we first analyzed data from these sleep stages based on this categorization.

In the same subjects, we also took advantage of the fact that both disconnected consciousness and unconsciousness intermittently occur in the same global brain state of NREM sleep, which allowed us to examine neural correlates of consciousness while controlling for the behavioral state^21^. The same subjects underwent a serial awakening paradigm whereby subjects were woken during the night by an alarm sound lasting 1.5 seconds. Subjects were also requested to inform us if they awoke during the TMS pulses. A structured questionnaire was conducted at the bedside. The first question was “Tell me everything that was going through your mind before the alarm sound/you woke up”. Participants were instructed to report whether they had any conscious experience, for example by delivering a dream report or report that they had had a conscious experience but did not remember its content (disconnected consciousness); or report that they had experienced nothing (unconsciousness). Further methodological details are available here^21^. To be clear, the NREM-sleep data from this serial awakening paradigm were then investigated in a further, separate analysis to confirm the findings from the “state-based” analyses.

### TMS data collection

Electrical brain activity was recorded using a 60-channel TMS-compatible EEG amplifier (Nexstim eXimia, Nexstim Plc, Finland) including a sample-and-hold circuit to prevent the amplifier from saturation. EEG was referenced to an additional frontal channel, filtered (0.1–350 Hz), and sampled at 1450 Hz. EEG channels were maintained with an impedance of less than 5 kΩ. After observing a minimum of three minutes of sleep, single-pulse TMS was delivered to the medial superior parietal cortex (superior parietal lobule and precuneus) using a Navigated Brain Stimulation system (eXimia NBS, Nexstim Plc, Finland) to ensure precise and reproducible stimulation. Specifically, a stereotactic infrared camera tracked the position and orientation of the coil with respect to the subject’s head and co-registration for the navigation was repeated during the night. The maximum electric field induced by TMS was focused on the convexity of the targeted cortical gyrus with the induced current perpendicular to it. The predominant stimulation direction was in the posterior–anterior direction. Using a figure-of-eight coil (Focal Bipulse, Nexstim Plc, Finland), biphasic TMS pulses were delivered at random intervals (2–2.3 seconds). To avoid auditory evoked potentials associated with the TMS-coil click, participants wore earphones with noise masking, and a thin foam pad was placed between the scalp and the coil. Between the TMS sessions, the coil was cooled using ice packs. The maximum electric field at the cortical target were similar across experiments: between 100 and 130 V/m for the sleep study and ~110 V/m for the anesthesia work. To minimize the risk that the subjects would wake up in the sleep experiment, TMS was started at 30–40% of the maximum stimulator output and increased in 10% increments every two stimulations, until the desired intensity was obtained. The TMS was discontinued with signs of arousal during the sleep-data acquisition.

### Preprocessing

The data were finite-impulse-response filtered at 0.5–40 Hz (EEGLAB) and epoched from −600 to 600 ms in SPM12. The data were manually cleaned through trial and channel rejection, including removal of trials with obvious pulse/electromyography/ringing or recharging artefact, and then bad channels were interpolated and EEG data were referenced to the average of all electrodes (SPM12). In the anesthesia experiments, the (mean [standard deviation]) number of trials across subjects for propofol was 202 [53] compared to 147 [48] for wakefulness and 185 [44] for ketamine compared to 149 [41] for wakefulness. In the sleep experiments, NREM (674 [320]) and REM (430 [282]) trials were compared to trials in wakefulness (545 [215]). In the serial awakening paradigm, there were 147 [85] trials during disconnected consciousness and 120 [58] during unconsciousness.

Time–frequency analysis in sensor space was then conducted for a group of 16 parietal and occipital electrodes overlying posterior cortex, as this area has recently been suggested to represent a posterior hot zone for cortical activity associated with consciousness^2,15^, (list C3, C1, Cz, C2, C4, CP3, CP1, CPz, CP2, CP4, P3, P1, Pz, P2, P4, and POz) using the following steps: data were first averaged across trials then across the selected electrodes. Time–frequency decomposition of averaged data was conducted using a Morlet wavelet transform response with seven wavelets, with baseline correction (−150 to −50 ms prior to TMS) performed in SPM12. Subsequently the data were entered into group-level T- or F-contrast analyses. For illustrative purposes, grand mean averages were created across subjects.

### Statistics

Initially we analysed the data by “state” comparing wakefulness to REM sleep or ketamine anesthesia (disconnected states) and propofol anesthesia or NREM sleep (unconscious states), using one-tailed T-contrasts with the hypothesized direction that alpha power would be greater in wake than in disconnected and unconscious states. Similar one-tailed hypotheses suggested that evoked high-frequency power would be diminished in unconscious states as per prior observations^6^. Although our hypotheses contained a specific directional contrast, *post hoc* we additionally checked for contrasts in the opposite direction and found no significant effects at p < 0.001. At this stage, we did not divide the NREM-sleep data based on the serial awakening paradigm. REM sleep and ketamine anesthesia were classified as disconnected states. NREM sleep and propofol anesthesia were classified as unconscious states. Our objective here was to identify the differences in TMS-evoked activity between well-recognized disconnected and unconscious states.

In a further confirmatory analysis, we analysed the NREM-sleep data sub-classified based on the reported conscious state in the serial awakening paradigm. Subjects were divided into disconnected consciousness and unconsciousness as previously^21^. Sensor-space time–frequency statistics of the evoked power were conducted in SPM12, with correction for multiple comparisons based on random field theory. Voxel based peak statistical thresholds were set at p < 0.05 corrected using Family Wise Error (FWE) over time (0–400 ms) and frequency (0.5–40 Hz in 1-Hz bins). Secondary analyses repeated the analyses in focused frequency windows (8–14 Hz in 1-Hz bins) and low-gamma band (30–40 Hz in 1-Hz bins). Our focus was on the reproducibility of our findings across states hence if a peak statistical FWE threshold was not achieved in an individual data set, the data were reported if they achieved the p < 0.001 uncorrected threshold. We also conducted focused analyses in the alpha (8–14 Hz) and low-gamma (30–40 Hz) bands based on *a priori* hypotheses and convention in the EEG literature. In general, however, most results reported in the present work survived p < 0.05 after FWE correction at the peak level.

## Results

### Evoked Alpha Power is Reduced in Disconnected Conscious States

**Figure 1:**
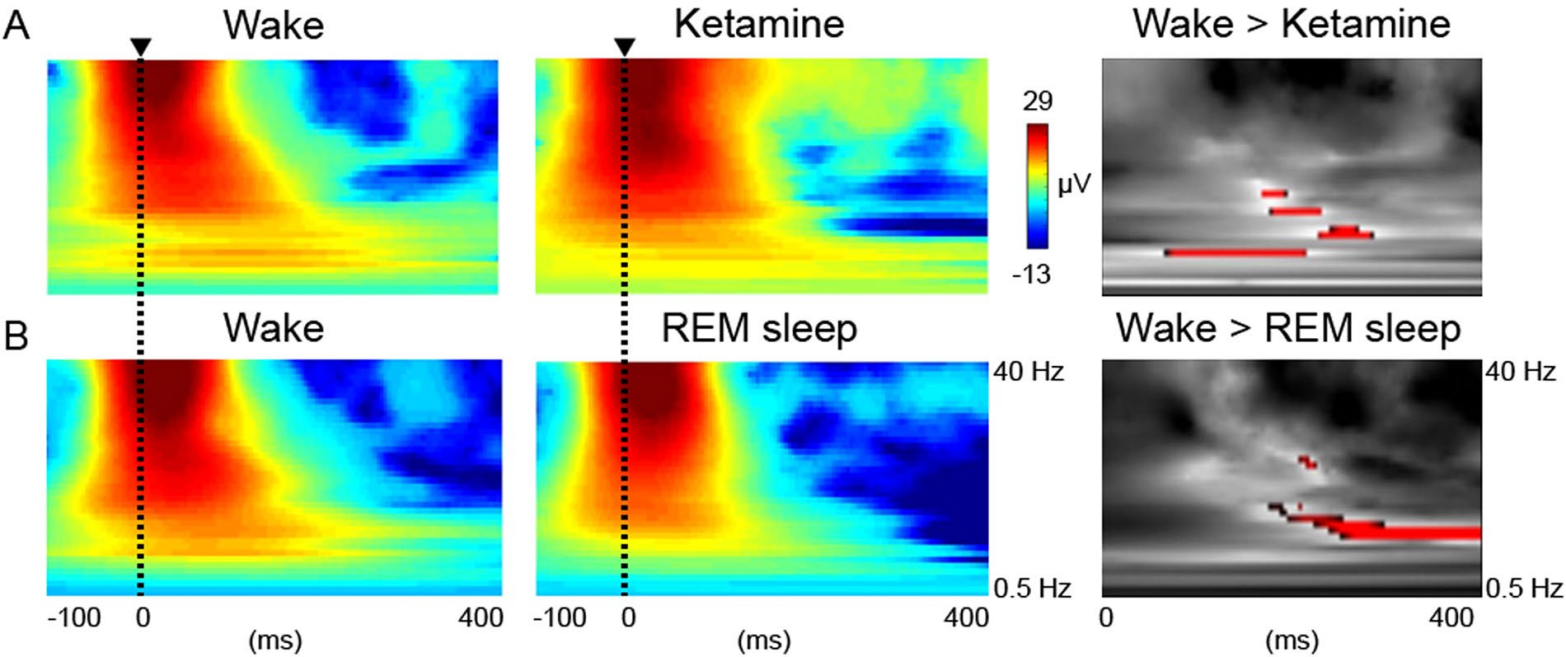
Sensory disconnection is associated with reduced evoked alpha power. (**A**) Subject-level grand mean averages of the posterior cortical response to TMS for wake (left) versus ketamine anesthesia (middle) displayed from −100 to 400 ms around the TMS pulse. T-contrast for significant responses in red (p < 0.001 for display purposes) for wake greater than ketamine anesthesia between 0.5–40 Hz and 0–400 ms (right). (**B**) Subject-level grand mean averages of the posterior cortical response to TMS for wake (left) versus REM sleep (middle). T-contrast for wake greater than REM sleep (right).

To assess whether alpha-band responses were reduced in disconnected conscious states following TMS, we performed time–frequency analysis on EEG data recorded over posterior cortex of data collected in wake compared to REM sleep (n = 7) or ketamine anesthesia (n = 6). Over the whole frequency range from 0.5–40 Hz, we identified significant decreases in alpha responses for REM sleep at 11 Hz (peak-effect FWE p = 0.012 at 336 ms; cluster-level FWE p < 0.001 11–14 Hz at 216–376 ms; Fig. 1). For ketamine anesthesia, no peak-effect responses survived FWE correction. However, responses were identified at p < 0.001 and cluster-level FWE significance at 8 Hz (peak-effect FWE p = 0.000, cluster-level FWE p = 0.005 8 Hz at 176 ms; Fig. 1) over the whole frequency range. When restricted to the alpha band from 8–14 Hz, we found significant differences between 8–11 Hz for ketamine anesthesia and 10–12 Hz REM sleep (peak-effect FWE p < 0.05).

### Evoked High-Frequency Power is Reduced in Unconscious States

**Figure 2:**
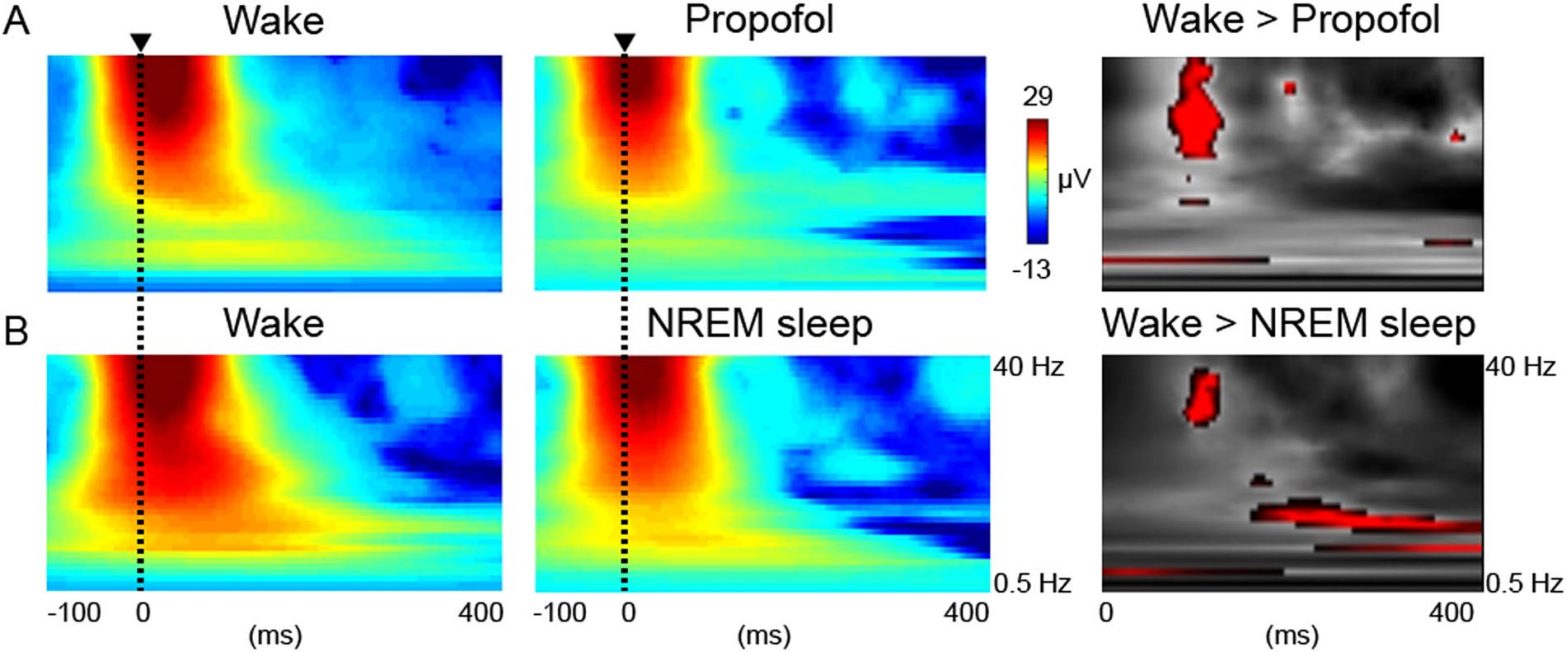
Unconsciousness is associated with suppression of evoked high-frequency power. (**A**) Subject-level grand mean averages of the posterior cortical response to TMS for wake (left) versus propofol anesthesia (middle) displayed from −100 to 400 ms around the TMS pulse. T-contrast for significant responses in red (p < 0.001 for display purposes) for wake greater than propofol anesthesia between 0.5–40 Hz and 0–400 ms (right). (**B**) Subject-level grand mean averages of the posterior cortical response to TMS for wake (left) versus NREM sleep (middle). T-contrast for wake greater than NREM sleep (right).

Next, we tested whether evoked low-gamma power was reduced in the unconscious states of propofol anesthesia and NREM sleep, compared to wakefulness. Evoked low-gamma power was decreased under propofol anesthesia and NREM sleep in a consistently narrow time window around 100 ms at 31 Hz (Fig. 2). Significant differences were apparent in the low-gamma band at 31 Hz (peak-effect FWE p = 0.006 at 100 ms; cluster-level FWE p < 0.001 31–36 Hz at 100–112 ms) for NREM sleep (n = 7) and 31 Hz (peak-effect FWE p = 0.005 at 100 ms; cluster-level FWE p < 0.001 31–38 Hz at 88–104 ms) for propofol anesthesia (n = 8). NREM sleep was additionally associated with decreased alpha-band responses at 13 Hz (peak-effect FWE p = 0.000 at 236 ms; cluster-level FWE p = 0.000 12–13 Hz at 236–320 ms) and 8 Hz (peak-effect FWE p = 0.005 at 400 ms; cluster-level FWE p = 0.003 8 Hz at 372–400 ms). In sensitivity analyses targeting the alpha (8–14 Hz) and low-gamma band (30–40 Hz) ranges, the same peaks were noted for NREM sleep (8–13 Hz and 31–36 Hz; all peak-effect FWE p < 0.05). The same analyses for propofol anesthesia revealed the same peak responses in the low-gamma band (31–38 Hz peak-effect FWE p < 0.05) but also significant differences in the alpha band (9 Hz peak-effect FWE p < 0.05). Importantly, we did not find changes in a focused analysis of the low-gamma band (30–40 Hz) for ketamine anesthesia or REM sleep (peak-effect FWE p > 0.05 or uncorrected p > 0.001), suggesting that evoked low-gamma activity is a signature of the conscious state.

### Across State F-contrast analysis of Time–Frequency Responses to TMS

**Figure 3:**
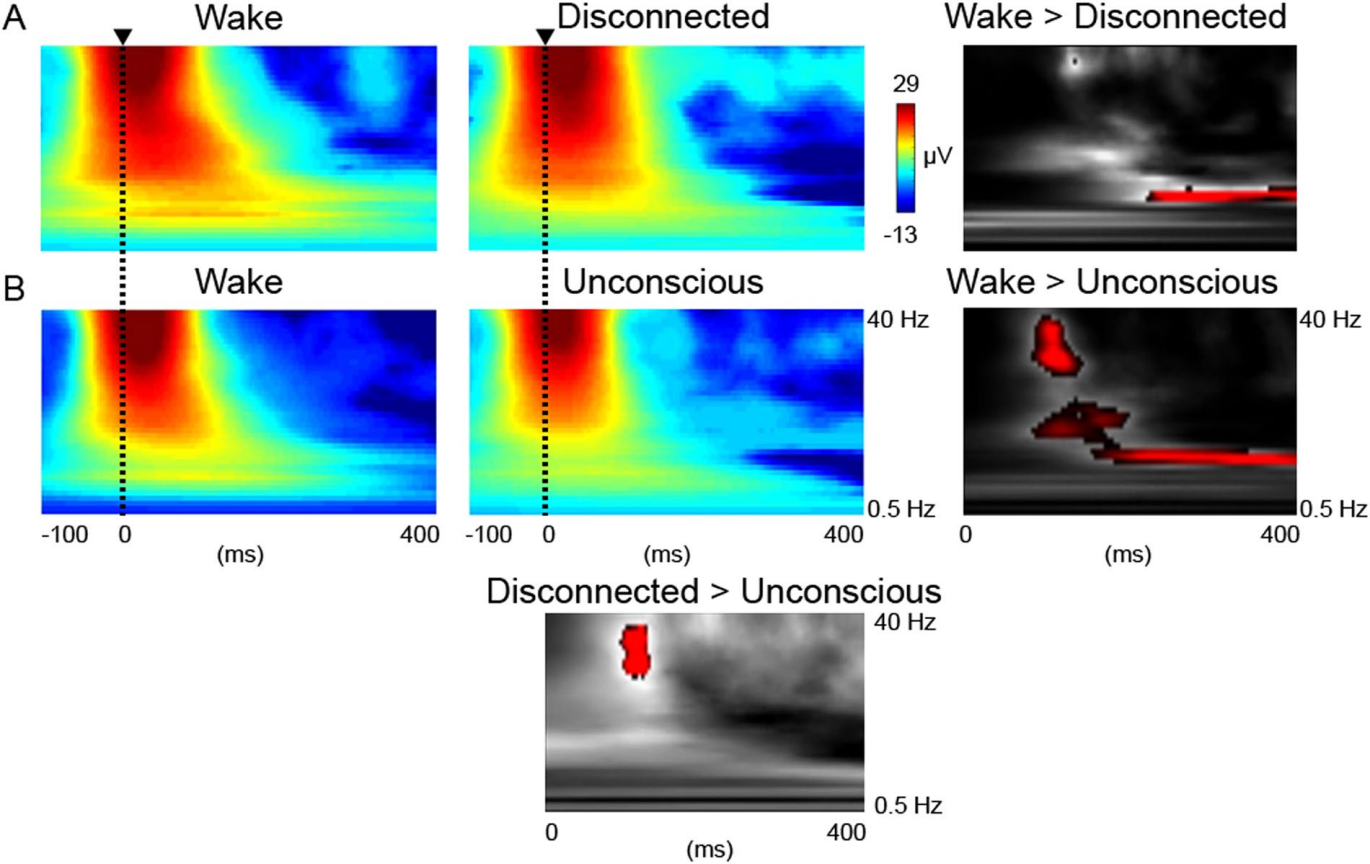
Unconsciousness and disconnected consciousness may be differentiated by evoked high-frequency power. (**A**) Group-level grand mean averages of the posterior cortical response to TMS for wake (left) versus disconnection (middle, REM-sleep and ketamine data combined) displayed from −100 to 400 ms around the TMS pulse. F-contrast for significant responses in red (p < 0.001 for display purposes) for wake greater than disconnected consciousness between 0.5–40 Hz and 0–400 ms (right). (**B**) Group-level grand mean averages of the posterior cortical response to TMS for wake (left) versus unconsciousness (middle, NREM-sleep and propofol data combined) displayed from −100 to 400 ms around the TMS pulse. F-contrast for significant responses in red (p < 0.001 for display purposes) for wake greater than unconsciousness (right). F-contrast for significant responses in red (p < 0.001 for display purposes) for disconnected consciousness greater than unconsciousness (bottom middle). There were no significant differences for unconsciousness greater than disconnected consciousness.

In summary, decreased evoked alpha power appeared as a signature of disconnected consciousness, while decreased low-gamma power was a correlate of the unconscious state. To confirm this, we conducted an F-contrast (ANOVA) analysis of the differences between wake and disconnected consciousness (REM sleep or ketamine anesthesia) or unconsciousness (NREM sleep or propofol anesthesia; Fig. 3). This analysis demonstrated that compared to wake, sensory disconnection was associated with decreased evoked alpha power with peak at 12 Hz (peak-effect FWE p < 0.001 at 400 ms; cluster-level FWE p < 0.001 12 Hz at 312–400 ms; Fig. 2) and also at 19 Hz (peak-effect FWE p = 0.014 at 168 ms; cluster-level FWE p = 0.010 11–14 Hz; Fig. 2). During unconsciousness the peak effects were at 12 Hz (peak-effect FWE p = 0.000 at 288 ms) and 31–35 Hz (peak-effect FWE p = 0.000 at 104–112 ms) that formed one cluster (cluster-level FWE p = 0.000 12–35 Hz at 104–288 ms; Fig. 1). Hence compared to wake, during unconsciousness, both decreased evoked low-gamma and alpha power were noted.

A further contrast was conducted that compared the states of disconnected consciousness (REM sleep or ketamine anesthesia) with unconsciousness (NREM sleep or propofol anesthesia). This demonstrated that the difference between these two groupings of conscious states involved decreased low-gamma power from 30 Hz at 112 ms (peak-effect FWE p = 0.003; cluster-level FWE p = 0.002).

### TMS responses in a serial awakening paradigm

**Figure 4:**
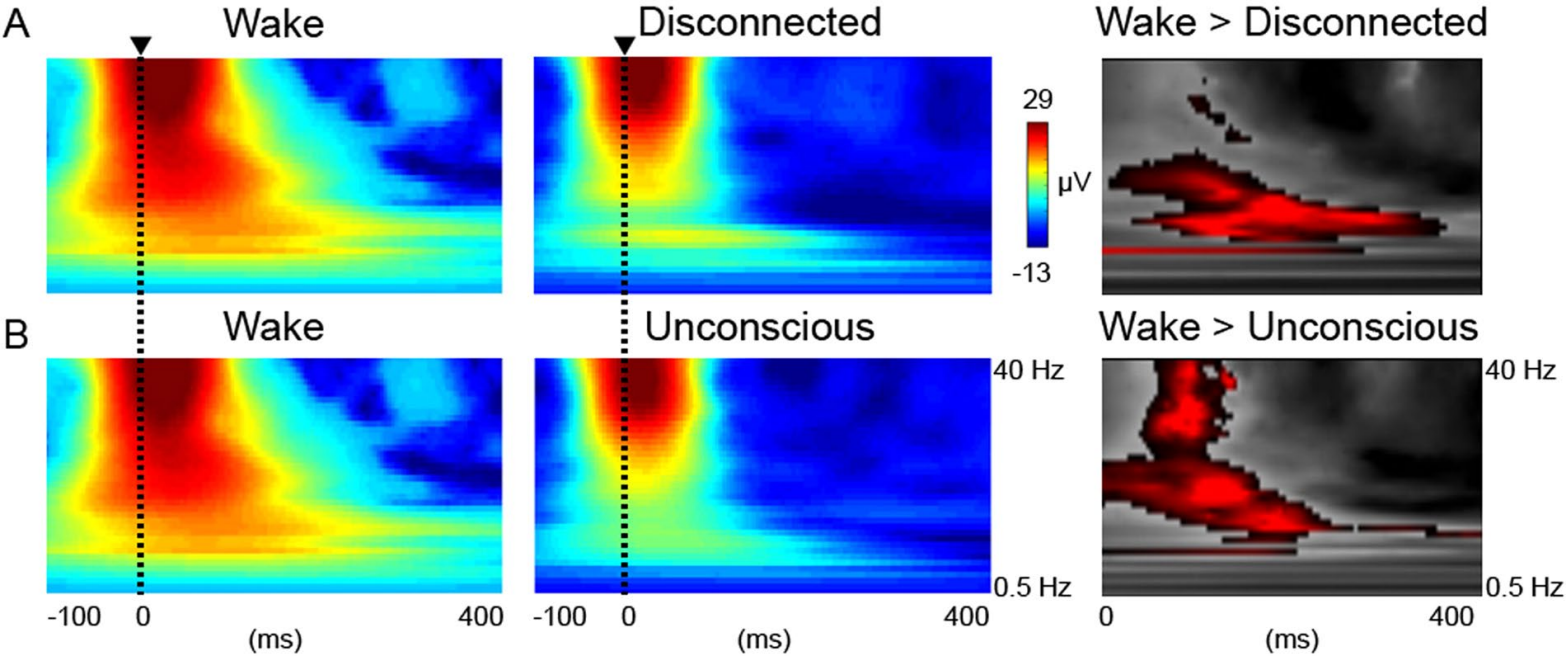
TMS responses in a serial awakening paradigm differentiate disconnected consciousness from unconsciousness during sleep. (**A**) Subject-level grand mean averages of the posterior cortical response to TMS for wake (left) versus confirmed disconnected consciousness during NREM sleep (middle) displayed from -100 to 400 ms around the TMS pulse. T-contrast for significant responses in red (p < 0.001 for display purposes) for wake greater than report-verified disconnected consciousness between 0.5–40 Hz and 0–400 ms (right). (**B**) Subject-level grand mean averages of the posterior cortical response to TMS for wake (left) versus confirmed unconsciousness during NREM sleep (middle). T-contrast for wake greater than confirmed unconsciousness during sleep (right).

Finally, we confirmed the above state-based analyses in the NREM-sleep data from subjects who were awoken from NREM sleep and asked to report their conscious state. Subjects were classified as having disconnected consciousness (n = 7) or unconsciousness (n = 7) based on the presence or absence of dream reports, respectively. TMS responses delivered in the 30 s before wake up were labelled in either dataset as per the previous analysis^21^ and then averaged at the subject level for the posterior electrodes. The mean (±standard deviation) number of TMS responses per subject was 116 ± 71 in the confirmed disconnected consciousness group and 75 ± 33 in the confirmed unconsciousness group. Group-level time–frequency analysis revealed that unconsciousness was associated with a peak change at 37 Hz (peak-effect FWE p = 0.023 at 136 ms; cluster-level FWE p < 0.001 18–33 Hz at 92–136 ms; Fig. 4). In contrast, for disconnected consciousness, we found no peak changes in the low-gamma band when analyzed over the whole frequency range or in a focussed analysis of the low-gamma band itself (30–40 Hz). However, in disconnected consciousness, evoked power in the alpha band was significantly reduced, with a peak at 13 Hz (peak-effect FWE p = 0.010 at 108 ms; cluster-level FWE p = 0.000 13–16 Hz at 108 to 180 ms; Fig. 4) and a separate peak at 8 Hz (peak-effect FWE p = 0.045 starting at 0 ms; cluster-level FWE p = 0.000; Fig. 4). Finally, we compared sensory disconnection with unconsciousness. Subjects who were unconscious had increased 14-Hz power at 228 ms compared to those who were disconnected (peak level FWE p = 0.055, cluster-level FWE p = 0.001, p < 0.001 uncorrected at 208–292 ms). In contrast, subjects who were disconnected showed increased 34-Hz power at 256 ms (peak level FWE p = 0.277, cluster-level FWE p = 0.362; p < 0.001 uncorrected) compared to those who were unconsciousness.

## Discussion

Compared to wake, TMS-evoked alpha activity was reduced in disconnected states of REM sleep and ketamine anesthesia. This finding was replicated in a subset of subjects in which dream reports were obtained following serial awakenings from NREM sleep. Together, these results suggest that the cortical and corticothalamic networks that generate alpha-oscillatory activity may be disrupted in disconnected states.

In the earliest EEG recordings, heightened alpha activity was apparent in the raw recording traces when awake subjects closed their eyes^23^. On the other hand, a decrease in spontaneous alpha activity is a classic signature that the subject is falling asleep, i.e., becoming disconnected from the environment^16^. In a recent study employing resting-state EEG, posterior alpha power proved a useful marker of anesthesia-induced loss and return of connected consciousness^17,24^. Nonetheless, the role of the alpha-band activity remains controversial^25,26^. A prominent view is that alpha activity may represent an idling rhythm acting to inhibit “offline” brain regions^26–28^. In contrast, a growing body of evidence suggests that alpha-band integration is important for perception of external sensory stimuli^26^. For example, the seminal study by Palva *et al*. showed that widespread phase-locked (evoked) alpha activity was necessary for perception of somatosensory stimuli^13^.

Why is evoked alpha power reduced in disconnected states? A recent study in non-human primates demonstrated that cortico-cortical interactions in the alpha range were partially coordinated by the pulvinar, a high-order thalamic nucleus^29^, that is well positioned to act as a gain control for sensory signals^30^. Thus, coordinated alpha activity involving higher-order thalamic nuclei may be required for the perception of sensory stimuli^13^. The pulvinar exhibits spontaneous alpha activity in wake^31^. Alpha activity decreases during REM sleep (along with high-frequency activity)^8^ and is replaced by slow waves, suggesting this nucleus may be “offline” during disconnected states. The pulvinar receives driving corticothalamic input, so it is possible that TMS in parietal areas could induce cortico-thalamocortical activity that may be reduced when the pulvinar is unable to act to increase cortical gain. Furthermore, alpha oscillations can help synchronize large populations of neurons, which may be important for the integration of conscious experiences^13,14,26^. Evoked alpha power may also reflect the recruitment of parietal hub regions that exhibit strong resting-state connectivity in the alpha band^14^. Parietal regions may contribute features of spatial awareness, loss of which may be critical to sensory disconnection. Given the widespread connectivity of the pulvinar, particularly between sensory and parietal areas, and the role of the alpha band in supporting the effective connectivity and function of these brain regions, future studies could focus on the disruption of this specific network as a possible substrate for sensory disconnection.

At this juncture, it is unclear if a common mechanism leads to disruption of evoked alpha in disconnected states or if multiple distinct mechanisms lead to the same observed outcome. Global patterns of electrical cortical activity are grossly similar in REM sleep and ketamine anesthesia; however, the neuromodulatory milieu associated with these states is distinct. During REM sleep, cholinergic tone is high but noradrenaline, serotonin, and histamine levels are low^32^. In contrast, ketamine anesthesia is associated with increased cholinergic^33–35^ and noradrenergic^36,37^ levels. It is possible that the lack of noradrenaline contributes to the mechanisms of disconnection in REM sleep^1^, while disconnection during ketamine anesthesia is produced by its effect on NMDA receptors^38^ or HCN1 channels^39^. Mechanistic studies interrogating the modulation of these factors on evoked alpha power are required.

We also found that unconsciousness during sleep and anesthesia was associated with reduced evoked low-gamma power following TMS of posterior cortex. This was apparent for NREM sleep and propofol anesthesia and occurred in a remarkably consistent time-frame of 100–125 ms (Fig. 2). The contrast of disconnected-conscious states with unconscious states (Fig. 3) and the analysis of wake compared to unconsciousness in the serial awakening paradigm (Fig. 4) also showed reduced low-gamma band evoked power. The remarkable consistency in the timing of gamma reduction is highly suggestive of a common mechanism. One likely possibility is that the loss in low-gamma power represents the occurrence of a synchronized neuronal down state and corresponding “OFF period”. Bistability between ON and OFF periods was observed with electrocortical stimulation in humans during NREM sleep^6^, which induces a loss of high-frequency activity with a similar latency as in the present data. The abrupt cessation of neuronal activity is thought to disrupt cortical effective connectivity and can account for the stereotypical responses to TMS observed in unconscious states. Conversely, the persistence of low-gamma activity in awake or disconnected patients may reflect the tonic activation of neurons in these states. Recent work^2–5,19,40–42^ suggests that the capacity for integrating information within a posterior cortical “hot zone” may be critical to the mechanism of consciousness. While our findings are broadly consistent with that suggestion, it is important to point out that we only stimulated posterior cortex and cannot exclude that similar results could be found with stimulation elsewhere, such as frontal cortex.

It is important to note that gamma oscillations have long been associated with consciousness^43^. Indeed 40-Hz synchrony has been hypothesized to play an important role in “binding” discrete stimulus attributes^44^. It is therefore of interest that gamma power was reduced following TMS during unconsciousness. Future studies should pursue the relationship with gamma synchrony (as opposed to power) as a signature of the conscious state. The relationship to high gamma oscillations is particularly important to explore, as coherence in this frequency range has been shown to be perturbed by the sleep states of REM and NREM and anesthetic states of ketamine, propofol and sevoflurane^45,46^. Ideally this will involve other approaches than TMS (due to possible contamination by electromyogram artefact) such as magnetoencephalography or electrocorticography.

This study was a secondary analysis of previous data. Our analysis was limited to TMS pulses applied in a single region, as we lacked data acquired across all states in different anatomical regions. Delivery of pulses at different cortical locations should determine (i) whether this effect is restricted to posterior cortex and (ii) whether it may be accompanied by an inversion in the main direction of activity flow, which has been hypothesized to occur in disconnected states^47^. In the analysis of the serial wake ups, we did not have enough data to divide disconnected consciousness with and without recall into separate states and future studies should investigate whether there are different signatures for these states. Furthermore in the serial awakening paradigm subjects who were unconscious had higher 14-Hz alpha power than when disconnected. This may mean that in the presence of consciousness, greater suppression of evoked alpha power, is required to maintain disconnection than in the state of unconsciousness. This is consistent with the relative sensory thresholds that are required to arouse subjects from REM sleep compared to N2 sleep. However we must stress that in the F-contrast across states we did not identify a more profound suppression of evoked alpha power in disconnected compared to unconscious states. Future dedicated studies should investigate whether evoked alpha power is more suppressed during disconnected consciousness compared to unconsciousness. It is also of interest that patients in the ketamine state reported hallucinatory dream-like experiences on recovery from the drug. Hence it is interesting that decreased alpha power has been noted in resting-state EEG analyses of subanesthetic ketamine when subjects have psychedelic experiences^48^. In this subanesthetic state, these subjects may be progressing to a state of disconnection, and this should be probed in future dosing studies with ketamine using resting-state EEG, sensory evoked responses and TMS. Other technical constraints should also be noted. A recent study has suggested that some of the TMS-evoked response may be driven by sensory stimulation rather than just direct cortical perturbation^49^. Given that the same stimulation parameters were used in both the wake and disconnected/conscious states it is likely that the relative contribution of transcranial and sensory responses was constant across the states. This simultaneously both supports that TMS may be used to study sensory disconnection (as it involves some sensory evoked component) and also the need for future studies that employ only sensory evoked responses to understand at what level of sensory processing disconnection can occur. We low-pass filtered our data at 40 Hz to reduce the impact of any electromyography artefact from the TMS pulse. Consequently, we cannot comment on higher frequencies that have been previously implicated in sensory perception^50^. Future studies could employ magnetoencephalography or electrocorticography to investigate the role of high-gamma activity. Also, our approach bypassed the ascending thalamus by stimulating the cortex directly with TMS. Given the crucial role of thalamo-cortical relays in sensory processing, future characterization of these relays using sensory stimulation in disconnected states is warranted. Future prospective studies could examine other anesthetics, notably dexmedetomidine, which also produce a disconnected state^51^. Finally, it is possible that our results are impacted by artefact from TMS pulses, despite our efforts to remove these from our dataset. However, we think such effects would be minor since our main effects are well beyond the time point when artefact is maximal. Also, physical artefact would be expected to be state-invariant and thus unlikely to affect our between-state comparisons.

In summary, in three different states of disconnected consciousness (ketamine ansethesia and REM and NREM sleep with report of conscious experiences), TMS-evoked alpha activity was reduced relative to the awake conscious state. These results suggest that a reduction of evoked alpha may represent a signature of sensory disconnection. Together with previous work showing a role for alpha activity in the perception of liminal stimuli in awake subjects, they point to the importance of the state of alpha-generating circuits in determining whether and how well a subject’s consciousness is connected to the environment. Further investigation of this hypothesis is needed to test (i) the utility of alpha as a marker of disconnection to various sensory stimuli (especially not limited to one sensory domain such as vision), (ii) the interaction with resting-state responses and (iii) the roles of different levels of the corticothalamic hierarchy in the generation of alpha across diverse states of consciousness and sensory disconnection. Determining the underlying cause of diminished alpha activity in disconnected states may yield important clues about sensory processing in general.
